# Prediction of Mind-Wandering with Electroencephalogram and Non-linear Regression Modeling

**DOI:** 10.3389/fnhum.2017.00365

**Published:** 2017-07-12

**Authors:** Issaku Kawashima, Hiroaki Kumano

**Affiliations:** ^1^Graduate School of Human Sciences, Waseda University Tokorozawa, Japan; ^2^Faculty of Human Sciences, Waseda University Tokorozawa, Japan

**Keywords:** support vector machine regression, machine learning, electroencephalogram, mind-wandering, neuro-feedback

## Abstract

Mind-wandering (MW), task-unrelated thought, has been examined by researchers in an increasing number of articles using models to predict whether subjects are in MW, using numerous physiological variables. However, these models are not applicable in general situations. Moreover, they output only binary classification. The current study suggests that the combination of electroencephalogram (EEG) variables and non-linear regression modeling can be a good indicator of MW intensity. We recorded EEGs of 50 subjects during the performance of a Sustained Attention to Response Task, including a thought sampling probe that inquired the focus of attention. We calculated the power and coherence value and prepared 35 patterns of variable combinations and applied Support Vector machine Regression (SVR) to them. Finally, we chose four SVR models: two of them non-linear models and the others linear models; two of the four models are composed of a limited number of electrodes to satisfy model usefulness. Examination using the held-out data indicated that all models had robust predictive precision and provided significantly better estimations than a linear regression model using single electrode EEG variables. Furthermore, in limited electrode condition, non-linear SVR model showed significantly better precision than linear SVR model. The method proposed in this study helps investigations into MW in various little-examined situations. Further, by measuring MW with a high temporal resolution EEG, unclear aspects of MW, such as time series variation, are expected to be revealed. Furthermore, our suggestion that a few electrodes can also predict MW contributes to the development of neuro-feedback studies.

## Introduction

Mind-wandering (MW; Smallwood and Schooler, 2006) can be defined as a thought that is irrelevant to the task or situation at hand preventing one from paying attention to the task/situation. While MW, is a common phenomenon, known to occupy 46.9% of daily life, it relates to various psychological problems. Through measurements in daily life, Killingsworth and Gilbert (2010) noted that happiness declines when a person is in MW. Further, the trends that can be observed with MW positively correlate with neuroticism, meaning difficulty in emotional regulation, alexithymia, and dissociation (Baer et al., 2006; Jensen et al., 2016). One study indicated that schizophrenia patients have a higher trait for MW and that the severity of positive symptoms and MW traits are correlated (Shin et al., 2015). In addition, the relationship between MW and anxiety has also been found, with a brain-imaging study proposing a model illustrating that trait anxiety strengthens MW mediated by the worrying trait (Forster et al., 2015). Furthermore, the relationship between depression symptoms and MW is particularly often investigated. Smallwood et al. (2007b) reported that people with high depressive traits experience MW more often than those with low traits. Other studies also reported the same result and indicated that MW frequency correlates with rumination traits (Burg and Michalak, 2010). Rumination is a crucial variable for the occurrence, maintenance, and deterioration of depression (Nolen-Hoeksema et al., 2008) and mediates between MW and symptoms of depression (Marchetti et al., 2014).

Therefore, MW is a critical theme for various psychiatric problems, including depression, and further research is needed. For investigating MW, it is important to know how to evaluate one's MW. Currently, to measure MW, many researches use thought sampling during a task. In this method, subjects complete a tedious task, such as a Sustained Attention to Response Task (SART; Robertson et al., 1997), and during the task, subjects report when that their mind wanders by button pressing. While this method is known as the self-caught thought sampling method, another available method is the probe-caught sampling, in which a thought probe asks subjects whether they are in MW through interrupting the task at several 10-s intervals (Smallwood et al., 2007a).

However, both methods have some limitations. First, the self-caught method is affected by the meta-awareness ability of subjects. People cannot generally monitor whether they are in MW, and awareness of MW is intermittent (Schooler et al., 2011). Thus, MW in subjects whose ability to realize their MW is weak can be hard to calculate. In addition, considering that a greater level of MW is disturbing to cognitive performance, the less likely one is to be aware of MW (Smallwood et al., 2008) as self-caught thought sampling is prone to failure in detecting higher levels of MW. Second, the probe-catching method is suitable for subjects with a shortage of meta-awareness (Smallwood and Schooler, 2006); however, the questions presented in this method inevitably interrupt subjects' MW.

Recently, reports predicting the existence of MW from biological multi-variance methods are increasing. Mittner et al. (2014) measured subjects' default mode network (DMN; Raichle et al., 2001) activity, known to be a neural basis of MW, by functional magnetic resonance imaging (fMRI) and the temporal changing of their pupil size. Mittner et al. (2014) then fitted a model estimating whether subjects were in MW with these measured data and a machine-learning algorithm. Bixler and D'Mello (2016) also succeeded in detecting MW during reading using oculometric variables, such as the changes in gaze direction and pupil size. These prediction models enable us to not only evaluate one's MW without an effect on either meta-awareness ability or questioning interruption but also quantify their MW with high temporal resolution.

However, these studies also have some limitations when evaluating MW. First, the models proposed by these studies only provide a binary estimation and are not able to refer to the “deepness” of MW. Previous studies claim that MW is not dichotomous but a phenomenon with continuous intensity (Schad et al., 2012; Farley et al., 2013). The intensity of MW can be an informative variance to research on MW. Allen et al. (2013) measured the intensity of MW during a task with thought sampling using a Likert scale and clarified the relation between the task performance and average and variance of intensity of MW. However, existing models just predict whether subjects are in MW or not. With a method of measuring continuous MW intensity with high temporal resolution, we can evaluate MW time series variation that has not been investigated previously, such as the sustaining duration of MW and the time it takes from noticing MW until returning to a concentrated state. Second, these models cannot be used in a general situation; installing and running fMRI device involves an enormous cost; eye-tracking devices are not applicable in the closed-eye state, such as trying to sleep or meditate (in many case they do it with eye-closed state), whose relationship to MW is getting attention (Drummond et al., 2013; Mrazek et al., 2013). Blanchard et al. (2014) tried to predict the existence of MW using skin conductance and temperature and to solve the problem of the model's low versatility. However, the said study remains imprecise, and the authors suggested the amount of information from skin conductance and temperature for prediction to be insufficient.

Electroencephalogram (EEG) measurement is easy, has few limitations in measurement circumstance, and expected to have an adequate amount of information. The EEG indicator can reveal the nature of MW in uninvestigated conditions, such as trying to sleep or meditate. Further, the EEG model is useful for neuro-feedback. If an EEG model requiring a short number of electrodes is obtained, a simplified portable EEG device can provide feedback to one's MW. The model may enable subjects to take mobile EEG feedback devices home like Zich et al. (2015) did and increase effectiveness through intensive home practice.

Some previous studies reported EEG features associated with MW, and many scholars investigated whether EEG changes represent DMN activity. Scheeringa et al. (2008), measuring fMRI and EEG spontaneously during the resting state, reported a negative correlation between theta frequency band (2–9 Hz) power on the frontal midline area and the BOLD intensity of the areas composing DMN. The relationship between EEG on the midline area and DMN activity has been indicated in other studies too. To investigate how DMN activities appear on EEG, Berkovich-Ohana et al. (2012) measured EEG before and after subjects transitioned from the resting state to the on-task state based on DMN's character activated in the resting state and inactivated in a condition demanding cognitive processing. Accompanying the transition from resting to on-task state, they observed decreased gamma (25–45 Hz) power on the midline area. The studies investigating EEG's association with MW also indicated the relations between midline EEG and MW. Braboszcz and Delorme (2011), performing self-caught thought sampling and EEG recording, observed decreasing theta (4–7 Hz) power on the parietal midline area and delta (2–3.5 Hz) power on the frontal midline area after subjects were aware of MW and concentrated on a task again. Considering these studies, although the reported frequency band is inconsistent, EEG changes of midline areas are possible representation of DMN activity.

In addition to midline areas, studies by Braboszcz and Delorme, (2011) and Berkovich-Ohana et al. (2012) indicated lateral prefrontal EEG changes. The report regarding frequency bands is, however, inconsistent; the former described gamma (25–45 Hz) power decreases with disappearing MW, whereas the latter beta (15–30 Hz) power increases. The lateral prefrontal cortex makes up the Executive-Control Network (ECN; Seeley et al., 2007). Usually ECN is activated in a contradictory way to DMN (Menon, 2011); however, when MW is deep and exists without meta-awareness, both ECN and DMN are activated in the same way (Christoff et al., 2009).

As discussed above, previous research implies that mainly the DMN and ECN domains relate to MW occurrence; however, considering the inconsistency of the frequency bands reported, EEG features indicate that the MW state may appear in a wide frequency area. Further, considering that the relation between ECN activity and MW varies according to the intensity of MW, the correlation between an ECN activity and MW may be non-linear.

In the present study, we aim to demonstrate what kind of regression model can estimate MW deepness. We hypothesize that multiple EEG variables combination predicts MW better than single variable considering the association of several brain areas and various frequency bands with MW. Furthermore, we propose that non-linear models are more suitable than linear ones owing to the complex relation between ECN and MW.

We fit some regression models, predicting the intensity of MW obtained from probe-caught thought sampling with multiple EEG variables and Support Vector machine Regression (SVR algorithm. SVR can advantageously deal with high dimensional data and provides not only a linear model but also a non-linear one. Few studies try to predict subjective reports from neural variables with SVR. Hoexter et al. (2013) created a model to predict a self-reported anxiety severity with MRI data and SVR and reported Pearson's correlation coefficient *r* = 0.49 as the predicting score. We expect that our best model also can parallel such preciseness. We examine the following hypotheses: the linear or non-linear multi-variate SVR model has significantly better accuracy than the linear single regression and that non-linear SVR model has significantly better accuracy than the linear SVR model. In this way, this study suggests that the combination of EEG variables and non-linear regression modeling can be a good indicator of MW intensity.

## Materials and methods

### Subjects

We called for participants using posters at Waseda University, and 50 people participated in the experiment. We set two exclusion criteria for the analysis: first, two people who scored more than 2 *SD* on a scale measuring the tendency for depression (details are described later) were removed from the sample. Second, five participants whose difference between reported maximum and minimum MW was under two points were excluded. All finally included subjects were right-handed; 21 were males and 22 females, and they averaged 21.77 (*SD* = 2.27) years.

The study was approved by the Waseda University Academic Research Ethical Review Committee, and all participants provided written informed consent.

### Procedure

After informed consent for participation was obtained, we assessed the subjects for depression symptoms by the Center for Epidemiologic Studies Depression Scale (CES-D; Radloff, 1977). This score was acquired to extract data from subjects who were suspected of having little meta-awareness, given the possibility of not collecting proper reports from participants with feeble meta-awareness even in probe-caught thought sampling. Some people with depression may not be able to describe their MW correctly during a task as weak meta-awareness of one's thoughts is a marked feature of depression (Segal et al., 2002).

We subsequently introduced three tasks for subjects and confirmed their understanding with some practice. After EEG electrodes were attached to them, they completed two tasks and rested for approximately 10 min; one task remained to be performed.

We acquired EEG data during three tasks. Each task included measures lasting 14 min before and after a 30-s resting state, except for the time of thought sampling and presentation of instructions. Task 1 required subjects to tap their finger, and task 2 was an oddball task. However, we do not report them in this article.

**Figure 1:**
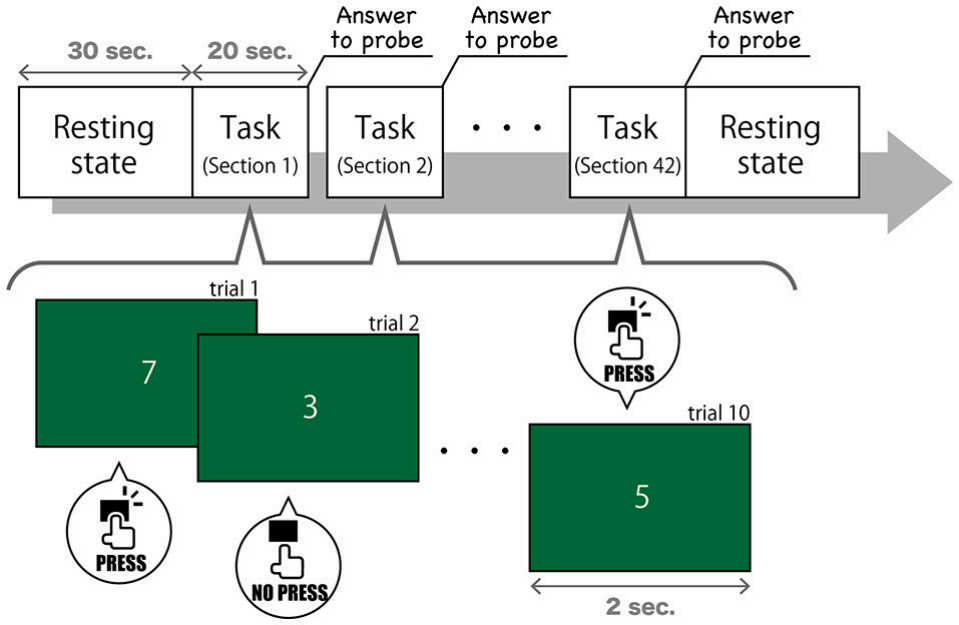
The procedure of Task 3.

Task 3, described in this study, modeled SART (Figure 1). In this task, the numerical digits 0–9 were presented on a screen in front of subjects in a pseudorandom order at 2-s intervals. Subjects were asked to press a button quickly when the number changed, but they were asked not to when the number “3” appeared. The number 3 was presented in 0.5% of the trials, which were composed of one number changing and one button pressing (or suspending to press) A thought sampling probe, inquiring where their attention was focused interrupted once every 20 s. We gave subjects the following instruction (in Japanese) to answer to the probe question on a 7-point Likert scale, ranging from “task-independent” to “task-centered”: “This question asks how much you have focused on the task. If you have been concentrating on the task, choose a lower number to rate. If your mind has wandered and you have thought about other things, choose a higher number to rate.” This probe models on Mittner et al. (2014), but the number of points had expanded from 5 to 7. Participants completed 42 sections, which were composed of 10 trials (20 s each) and one thought probe. Hence, we got 840 s of recorded data of EEG and 42 answers to probe. Considering the substantial variability between subjects for their MW rating, we normalized the reported MW scores in each subject.

### Behavioral data

To confirm that the reports of the MW intensity are valid, we investigated whether the behavioral data and off-task reports correlated. We adopted a static method that was used in a previous study (Kucyi et al., 2016) demonstrating that the variance of reaction time (RT) to a cognitive task correlates positively to the self-reported intensity of MW. First, we calculated Pearson's correlation between the variance of RT in each 42 sections and reported MW score within each subject. Then, we converted acquired *r* value into Fisher's *z* and applied a Wilcoxon signed rank test to them.

### EEG recording and preprocessing

We recorded the EEG data using the Geodesics EEG system (Electrical Geodesics Inc.) and 17 electrodes (F3, F4, F7, F8, Fz, T3, T4, TP9, TP10, P5, P6, P9, P10, Pz, O1, O2, and Oz), with a 250 Hz sampling rate, referenced to the Cz electrode. Impedance was kept under 50 kohm as per the recommendation of Electrical Geodesic Inc. We filtered the data using a 0.3–70 Hz band-pass filter and a 50 Hz notch filter. This filtering process was completed using Waveform Tools, Net Station Version 4.2 (Electrical Geodesics Inc.).

The EEG data was divided into 1-s epochs, and the ones contaminated with artifacts, such as eye movement, blinks, and body movement, were removed. The artifact detection algorithm was the same as that provided by Waveform Tools. All epochs were Fourier-transformed, and the mean power value and the coherence between each pair of electrodes in eight frequency bands (Kubicki et al., 1979; delta: 1.5–6.0 Hz, theta: 6.5–8 Hz, alpha1: 8.5–10 Hz, alpha2: 10.5–12 Hz, beta1: 12.5–18 Hz, beta2: 18.5–21 Hz, beta3: 21.5–30 Hz, gamma: 35–44 Hz) were calculated. These values were averaged in each section and normalized in each subject.

We then divided all participants into two groups: one provided training dataset, and the other provided test dataset including those of one-third of all subjects who were not used for model construction but for verification. Finally, we removed the sections in which any of the EEG data (i.e., the mean power value and the coherence) having a Z-score more than 5 was clubbed as an outlier. Using the abovementioned process, we obtained training dataset including 440 data samples and test dataset including 187 data samples (note that many sections were totally contaminated by artifacts and removed.) One data sample included 1,224 predictors [(17 electrodes for power values + _17_C_2_ electrodes pairs for coherence) × 8 frequency bands] and one response variable, indicating the intensity of MW, i.e., the target to predict. Both datasets were scaled by the average and variance of training dataset.

### Predictor selection

As the collected data included too many predictors and due to concern regarding over-fitting (severe deterioration of prediction accuracy when a model is applied to novel datasets), the predictors needed to be selected. The current study employed a filter technique with Pearson's correlation coefficient, which is applicable to the model fitting the algorithms we used. We screened out predictors whose absolute value of correlation coefficient |*r*| to the response variable was below a threshold (Mwangi et al., 2014). We prepared some predictor-set patterns using thresholds ranging from 0 to the maximum |*r*| with 0.01 intervals. Furthermore, to estimate the predictive accuracy of single variable regression, we added a predictor-set pattern including only one predictor that showed the highest |*r*| value. We calculated these correlation coefficients only with the training dataset.

### Model fitting

Support Vector machine Regression (SVR) is based on a linear regression function:

(1) $$f\left(x_{i}\right)\;=\;{x_{i}}^{T}\beta +\;\beta_{0}$$

*x*_*i*_ denotes *i*-th sample of datasets and β is the weight vector. To optimize weight vector, SVR minimizes the problem:

(2) $$H\left(\beta \right)=\;C\sum_{i\;=1}^{N}V\left(y_{i}-f\left(x_{i}\right)\right)+\;\frac{1}{2}\|\beta {\|}^{2}$$

where,

(3) $$\begin{array}{l} V_{H}\left(r\right)\;=\;\{\begin{array}{c} 0\;if\;\left|r\right|<\;\epsilon ,\\ \;\left|r\right|-\;\epsilon ,\;otherwise. \end{array} \end{array}$$

*y*_*i*_ is *i*-th response variable and *N* is 440 in current study. ϵ and *C* are user defined parameters. The model ignores the prediction error lesser than ϵ. *C* regulates trade-off balance between error smallness and model simplicity and contributes to avoid over-fitting. By replacing dot product *x*_*i*_, *x*_*j*_, which is used to solve above problem (for detail, see Hastie et al., 2009), by a kernel function *k*(*x*_*i*_, *x*_*j*_), SVR provides non-linear regression models. The present study used linear SVR and Radial Basis Function (RBF) kernel SVR:

(4) $$k\left(x_{i},\;x_{j}\right)\;=\;exp\left(-\gamma \|x_{i}-\;x_{j}{\|}^{2}\right)$$

γ is also user defined parameter and regulates model simplicity.

We determined ϵ, C, and γ using a grid search, which tries all patterns of parameter candidates to make models and adopts the best prediction accuracy combination. The grid search method uses cross-validation for the presumption of precision. In this approach, training datasets are divided into some (in this study: 10) groups in as equally as possible; one group is set as the test dataset in cross-validation; and the others are set as training dataset in cross-validation. After a model fitting with training dataset in cross-validation, we evaluated the mean squared error (*MSE*) between measured values and estimated values with test dataset in cross-validation. Another group was then assigned as test dataset in cross-validation, and *MSE* was revealed by the same procedure. This operation was repeated until all groups had been set as test dataset in cross-validation, and finally, the average of all *MSEs* was regarded as the estimated prediction score of the parameter pattern.

We applied single regression analysis to the training dataset of the single predictor pattern and two SVR algorithms to the other predictor-set patterns and estimated each cross-validation *MSE* score. We adopted predictor-patterns producing the best score, and additionally, the best one among the patterns using ≤9 electrodes considering model's usefulness. Thus, we fitted five models. Models 1 and 2 are non-linear SVR models using RBF, Models 3 and 4 are linear SVR models, Models 2 and 4 use a few (under 10) electrodes and are expected to have good versatility, and Model 5 is single regression model. We applied these models to the test dataset and obtained correlation coefficients between estimated values and measured values as that is indicative of the model's precision.

While Model 1 was expected to provide as high a level of accuracy as possible and meet the demands of basic MW research, Model 2 was adapted to situations using limited-measurement environments, such as neuro-feedback at home and expected to show less but close score to Model 1. Models 3 and 4 were created to examine if non-linear model predicts MW more precisely than linear model, and Model 5 confirmed multiple regression models (Models 1–4) as it had better precision than previously proposed single regression models. For these comparisons, we examined the significant difference in *r*-values between models: Models 1 vs. 2, Models 3 vs. 4, Models 1 vs. 3, Models 2 vs. 4, Models 1–4 vs. Models 5. We performed the above analysis using MATLAB R2016a (MathWorks).

## Results

First, to provide the validation of the reports of MW intensity, we used a Wilcoxon signed rank test. The mean within-subject correlation between RT variance and reported MW intensity was positive and significantly >0 (*p* = 0.000060).

**Figure 2:**
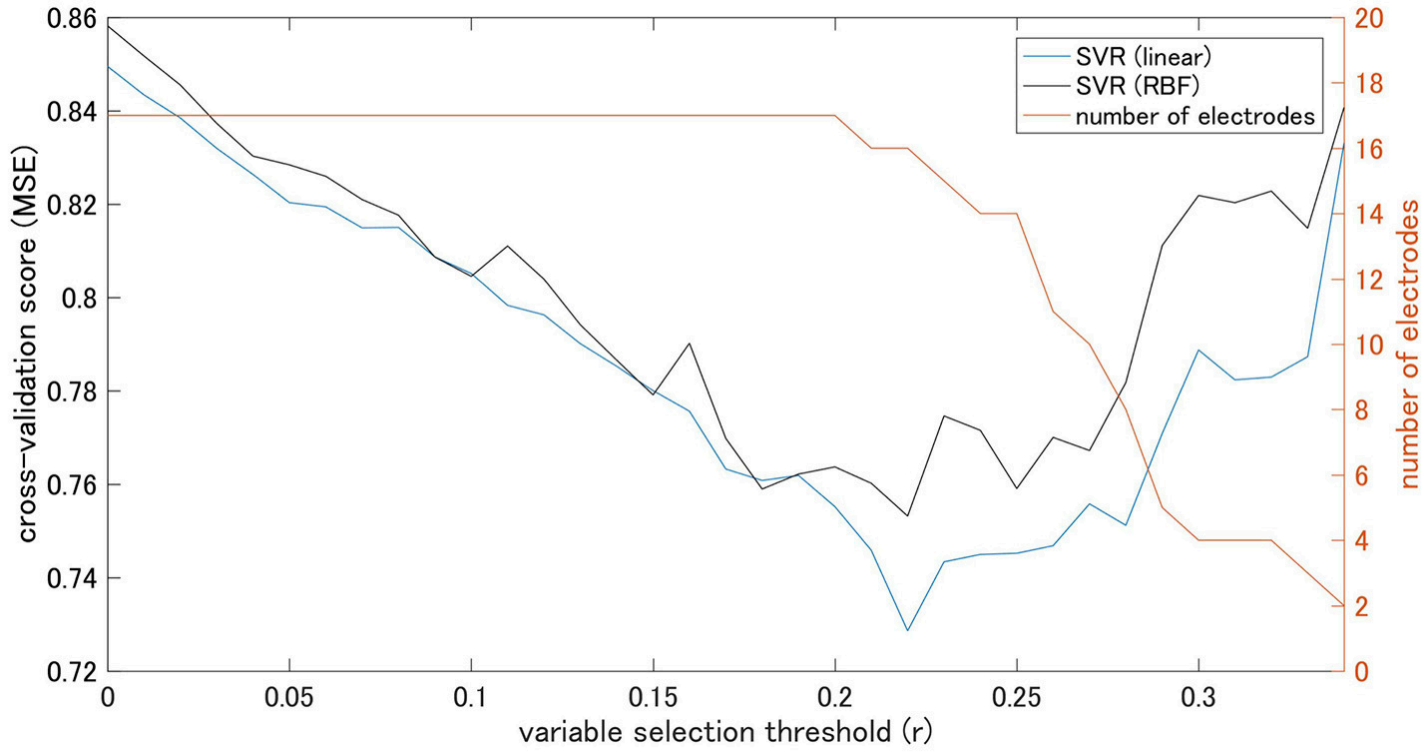
Cross-validation scores of Support Vector machine Regression (SVR) models on each threshold and number of electrodes.

**Figure 3:**
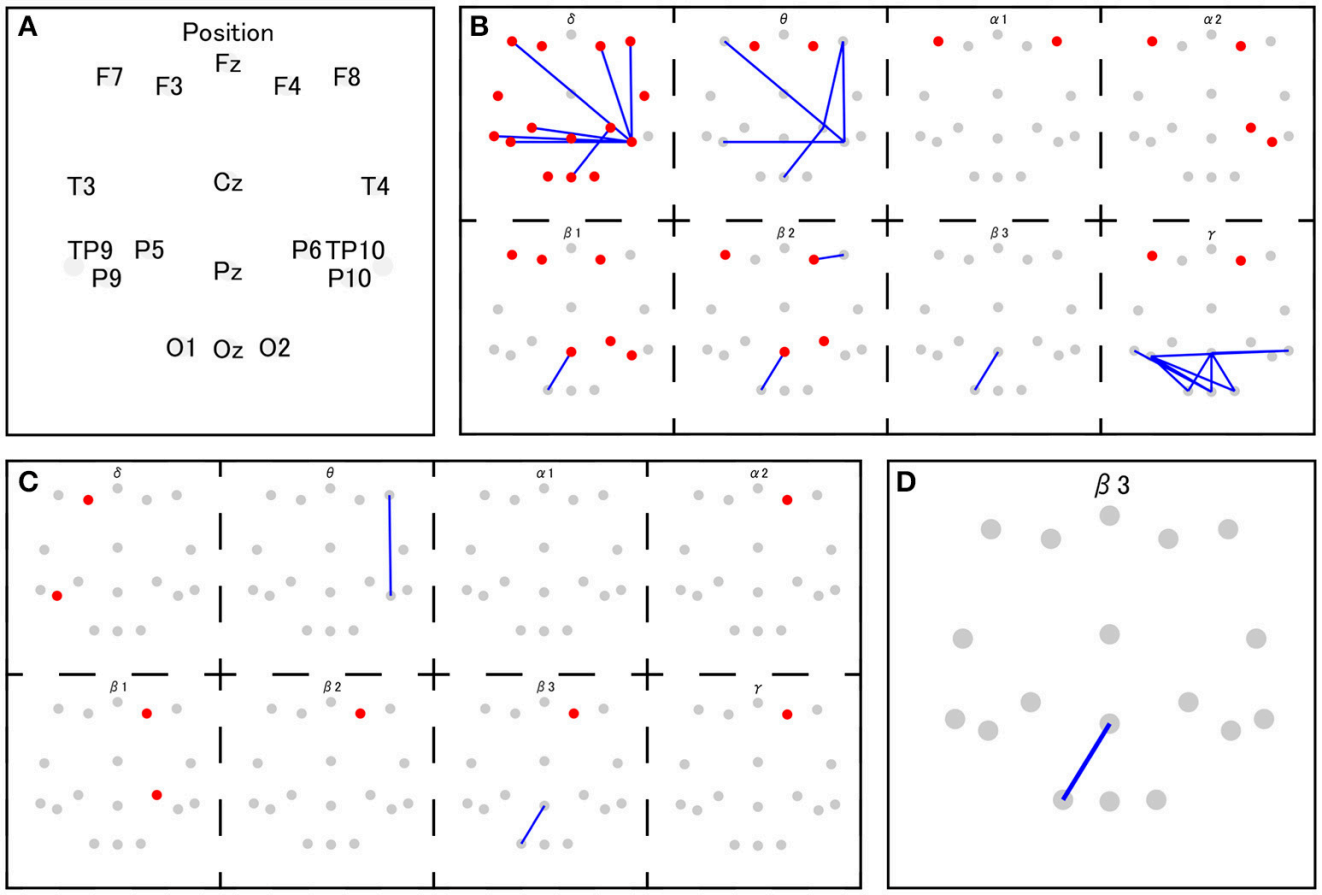
The set of selected features in Model 1 and 3 **(B)**, Model 2 and 4 **(C)**, and Model 5 **(D)**. The red and gray dots indicate disposed electrodes, and **(A)** indicates their corresponding names. The red dots mean that the power value of that electrode is used as a feature. The blue lines mean the coherence between those two electrodes is used as a feature (e.g., **D** indicates that the coherence between Pz and O1 in beta 3 frequency band is used as a feature in Model 5).

The coherence between electrode Pz and O1 in the beta-3 band showed the strongest correlation with response values (|*r*| = 0.346), and thus 35 patterns of predictor-set with thresholds ranging from |*r*| = 0.00 to 0.34 were acquired. We presumed the accuracy of linear and non-linear SVR models when each patterns are adopted by cross-validation. Consequently, the predictor-set with threshold |*r*| = 0.22 showed the best score in both non-linear and linear algorithms and we fitted Models 1 and 3 from this predictor-set. Seven predictor-set patterns using under 10 electrodes were acquired, and the predictor-set with threshold |*r*| = 0.28 indicated the lowest *MSE* in both non-linear and linear algorithms and were set as predictors of Models 2 and 4. These models used eight electrodes (F3, F4, F8, P6, P9, P10, Pz, and O1), while Models 1 and 3 used 16 (F3, F4, F7, F8, T3, T4, TP9, TP10, P5, P6, P9, P10, Pz, O1, O2, and Oz). We illustrate *MSE* and the number of used electrodes in each of the 35 predictor-sets in Figure 2 and list them with the value of three grid searched parameters: γ, ε, C in Table 1. We illustrate variables finally chosen in five models in Figure 3.

**Table 1:** The *MSE*, the number of used electrodes, and used parameters for each threshold and algorithms.

**Threshold (Pearson's *r*)**	**Number of electrodes**	**SVR (RBF)**				**SVR (linear)**			
		***MSE***	**γ**	**ε**	**C**	***MSE***	**γ**	**ε**	**C**
0.000	17	0.858	0.0005	0.500	0.500	0.850	0.0005	0.250	0.125
0.010	17	0.852	0.0005	0.500	0.250	0.843	0.0005	0.250	0.125
0.020	17	0.846	0.0005	0.500	0.250	0.838	0.0005	0.250	0.125
0.030	17	0.837	0.0005	0.500	0.250	0.832	0.0005	0.250	0.125
0.040	17	0.830	0.0005	0.500	0.250	0.826	0.0005	0.250	0.125
0.050	17	0.828	0.0010	0.500	0.250	0.820	0.0010	0.250	0.063
0.060	17	0.826	0.0010	0.500	0.250	0.819	0.0010	0.250	0.063
0.070	17	0.821	0.0010	0.500	0.250	0.815	0.0010	0.250	0.063
0.080	17	0.818	0.0010	0.500	0.125	0.815	0.0010	0.250	0.063
0.090	17	0.809	0.0010	0.500	0.125	0.809	0.0010	0.500	0.125
0.100	17	0.805	0.0010	0.500	0.125	0.805	0.0010	0.500	0.125
0.110	17	0.811	0.0020	0.500	0.125	0.798	0.0020	0.500	0.063
0.120	17	0.804	0.0020	0.500	0.125	0.796	0.0020	0.500	0.063
0.130	17	0.794	0.0020	0.500	0.125	0.790	0.0020	0.500	0.063
0.140	17	0.787	0.0020	0.500	0.125	0.785	0.0020	0.500	0.063
0.150	17	0.779	0.0020	0.500	0.125	0.780	0.0020	0.500	0.125
0.160	17	0.790	0.0039	0.500	0.125	0.776	0.0039	0.500	0.063
0.170	17	0.770	0.0039	0.500	0.125	0.763	0.0039	0.500	0.063
0.180	17	0.759	0.0039	0.500	0.125	0.761	0.0039	0.125	0.063
0.190	17	0.762	0.0039	0.500	0.125	0.762	0.0039	0.500	0.063
0.200	17	0.764	0.0078	0.500	0.125	0.755	0.0078	0.250	0.063
0.210	16	0.760	0.0078	0.500	0.125	0.746	0.0078	2.000	2.000
**0.220**	**16**	**0.753**	**0.0078**	**0.500**	**0.125**	**0.729**	**0.0078**	**2.000**	**4.000**
0.230	15	0.775	0.0156	0.500	0.125	0.743	0.0156	2.000	2.000
0.240	14	0.772	0.0156	0.500	0.125	0.745	0.0156	0.016	0.063
0.250	14	0.759	0.0156	0.500	0.125	0.745	0.0156	0.250	0.063
0.260	11	0.770	0.0313	0.250	0.125	0.747	0.0313	0.125	0.063
0.270	10	0.767	0.0313	2.000	4.000	0.756	0.0313	0.125	0.063
**0.280**	**8**	**0.782**	**0.0625**	**0.500**	**0.250**	**0.751**	**0.0625**	**2.000**	**1.000**
0.290	5	0.811	0.1250	0.500	0.063	0.771	0.1250	2.000	1.000
0.300	4	0.822	0.1250	1.000	0.250	0.789	0.1250	1.000	0.125
0.310	4	0.820	0.2500	1.000	0.500	0.782	0.2500	1.000	0.063
0.320	4	0.823	0.2500	2.000	1.000	0.783	0.2500	1.000	0.250
0.330	3	0.815	0.5000	0.500	0.250	0.787	0.5000	0.500	0.125
0.340	2	0.841	1.0000	0.500	0.125	0.833	1.0000	0.500	0.500

*The values with which the models are fitted are indicated by bold style*.

*MSE, Mean square error; RBF, Radial basis function; SVR, Support vector machine regression*.

We then investigated the precision of the five models with test dataset. The correlation coefficients between estimated values and measured values were *r* = 0.54 in Model 1, *r* = 0.49 in Model 2, *r* = 0.51 in Model 3, *r* = 0.39 in Model 4, and *r* = 0.35 in Model 5. To compare accuracy of the models, the differences in *r*-values were examined. Comparison between Models 1–4 and Model 5 (multiple predictors vs. single predictor) results significant differences in *r* between Models 1–3 and Model 5 (vs. model 1: *Z* = 3.45, *p* = 0.00026; vs. model 2: *Z* = 2.45, *p* = 0.0067; vs. model 3: *Z* = 2.53, *p* = 0.0057) but not between Model 4 and Model 5 (*Z* = 0.56, *p* = 0.29). All models' accuracy and difference in *r*-values compared to Model 5 are summarized in Table 2. Comparison between Models 1 and 3 and Models 2 and 4 (non-linear vs. linear) revealed that significant differences in *r*-values existed between Models 2 and 4 (*Z* = 3.49, *p* = 0.00024) but not between Models 1 and 3 (*Z* = 1.08, *p* = 0.14). Comparison between Models 1 and 2 and Models 3 and 4 (full electrodes vs. limited electrodes) revealed that significant differences existed between Models 3 and 4 (*Z* = 2.98, *p* = 0.0014) but not between Models 1 and 2 (*Z* = 1.27, *p* = 0.10). These results are summarized in Table 3.

**Table 2:** The results of Pearson's correlation test and *r* difference test between Model 5 and Model 1–4.

	**Pearson's correlation test**		***r* differ test (vs. model 5)**	
	**|*r*|**	***p***	***Z***	***p***
Model 1 (RBF, full electrodes)	0.54	1.02E-15	3.47	0.00026
Model 2 (RBF, limited electrodes)	0.49	8.40E-13	2.48	0.0067
Model 3 (linear, full electrodes)	0.51	9.74E-14	2.53	0.0057
Model 4 (linear, limited electrodes)	0.39	4.79E-08	0.56	0.29
Model 5 (single electrodes)	0.35	1.18E-06	–	−

*RBF, Radial basis function*.

**Table 3:** The result of *r* difference test.

	***Z***	***p***
Model 1 vs. Model 3 (full electrodes, RBF vs. linear)	1.08	0.14
Model 2 vs. Model 4 (limited electrodes, RBF vs. linear)	3.49	0.00024
Model 1 vs. Model 2 (RBF, full vs. limited electrodes)	1.27	0.10
Model 3 vs. Model 4 (linear, full vs. limited electrodes)	2.98	0.0014

*RBF, Radial basis function*.

## Discussion

The aim of this study was to prove that a model with multiple EEG variables and non-linear regression estimated MW intensity better than single variable or linear models. First, we confirmed that the RT variance correlates to self-reported MW intensity as shown in previous research and validated the reported MW score. Then, we prepared a combination of patterns of predictors and fitted models by SVR. Finally, using prediction accuracy estimated by cross-validation and the number of used electrodes, we proposed five models: Models 1 and 2 are non-linear models, Models 3 and 4 are linear models, Models 2 and 4 use restricted number of electrodes, and Model 5 is a single regression model. All models showed robustness, and Model 1–3 presented higher accuracy than similar SVR-using studies (Hoexter et al., 2013) through examination using held-out test data.

The variable indicating the highest correlation coefficient with response values was the beta 3 coherence between the parietal midline area (Pz) and the occipital area (O1). Previous research suggests that EEG over the midline area reflects DMN activity (this variable is suspected to relate to DMN). In addition to this variable, Models 2 and 4 include beta 1 and beta 2 activities over the lateral prefrontal area and beta 1 EEG over the parietal area. Considering that both areas are known to be a part of the ECN (Seeley et al., 2007), Models 2 and 4 might handle the information on ECN activity. Models 1 and 3 markedly add to coherence between the prefrontal area and right parietal area on low-frequency bands. The same coherence pattern has been reported in previous research, in which this EEG network, observed during mental arithmetic, correlated with BOLD signals from the right pre-motor area, left cerebellum, and left angular gyrus (Mizuhara and Yamaguchi, 2007). The pre-motor area and cerebellum are known to attribute to the preparation and coordination of physical movements (Ito, 2000; Schubotz and von Cramon, 2003). Moreover, the authors interpreted the observed EEG activity as a reflection of visual imaging of numbers, based on the previous research in which similar pattern of coherence was reported during the manipulation of abstract visual patterns (Sauseng et al., 2005) Thus, characteristic coherences in Models 1 and 3 may also be associated with the preparation for button pressing or the processing of the visual image of presented numbers.

Significantly higher accuracies of Models 1–4 compared to Model 5 partially indicate the validity to use multi-variate regression algorithms for estimation of MW intensity from EEG data. Models 1 and 3 showed no significant differences in their precision, and the suitability of the non-linear regression algorithm over the linear one was not confirmed. However, when the number of electrodes was limited, the non-linear model (Model 2) indicated better accuracy than the linear model (Model 4). It is seemed that Model 5 predicted the intensity of MW from DMN activity, and Model 2 and 4 used additional ECN activity. The Non-linear relationship between ECN activity and MW could be the possible cause for better accuracy in Model 2 than in Model 5, but not in Model 4. However, Models 1 and 3 indicated significantly higher precision than Model 5 since these models seemed to additionally use the brain activity involving pressing the button or the processing of numbers for prediction, and importance of ECN activity for prediction might be relatively small for them.

This research has some limitations. First, all subjects were young (averaging 21.77 years), and it is not clear whether the proposed models work on older people. Previous research indicates that aging decreases MW frequency during tasks. Zavagnin et al. (2014) investigated the MW frequency of several age groups using SART and probe-caught thought sampling, showing that MW reports reduce as age increases. As physiological support, Damoiseaux et al. (2008) reported that DMN activity during the resting state decreases in older subjects. However, it is unlikely that neural mechanisms of MW occurring in older people are qualitatively distinct from that in younger people as Maillet and Schacter (2016) explained that age-related differences in task interest cause this contrast. Hence, we assume that the present results are reproducible in older subjects, although further research is needed to establish this. Second, whether our models apply to EEG data during other conditions is unclear. We used EEG data during SART to make models requiring simple vigilance, and they may fit EEG in various tasks. However, in this experiment, frequent presentation of thought probes could make subjects conscious of MW and enhance their meta-awareness to MW. Further, the intervals of probes were fixed and the possibility that some subjects anticipate the probe occurrence timing cannot be denied. These influences of the presence of thought probe to EEG patterns may deteriorate prediction accuracy when the models were applied to conditions which have no probes. Further, Models 1 and 3 seem to be estimated on the basis of the neural activity for processing numerical visual images and button-pressing and may not be applicable to other conditions. Conversely, although further research is required, we implied that Model 2 uses only activity of ECN and DMN and can estimate MW in diverse settings.

As advanced research, a prediction model focused on the MW with the strict definition is worth investigating. The present study used probes including a questioning probe asking where the attention was focused on. The same probes were used in previous researches in which prediction model was created from physiological measures (Blanchard et al., 2014; Mittner et al., 2014; Bixler and D'Mello, 2016). However, recent researches (Stawarczyk et al., 2011; Smallwood and Schooler, 2015) suggest that “task-independent” or “off-task” answer can indicate various conscious states, such as a state distracted by external stimulus (e.g., sound of measurement devices), guessing the purpose of the task, or MW in a narrow sense (self-generated thought that is totally irrelevant to the task). By assessing the content of thoughts and purifying the target of prediction, the accuracy of prediction may improve. However, many studies, like the present study, regard all the states that are not attentive to the current task as MW (e.g., Mason et al., 2007; Christoff et al., 2009; Jensen et al., 2016; Maillet and Rajah, 2016). Particularly, such MW in the broad sense has recently been deemed to be an intervention target psychiatric problem (Segal et al., 2002; Kabat-Zinn, 2015). A proper prediction target depends on the purpose.

In conclusion, we illustrated that non-linear regression algorithm with multiple EEG variables estimates MW intensity well. A prediction by EEG enabled us to evaluate intensity of MW in high temporal resolution and observe uninvestigated aspects of MW, such as time-series variation. Moreover, although future research is required, MW estimation by EEG might be applicable to various situations. Our proposed method is expected to clarify the nature of MW in various little-examined situations, such as those involving attempts to sleep or meditate. Further, we demonstrated that EEG data from a few electrodes can also precisely estimate the intensity of MW and contribute to the development of neuro-feedback studies.

## Ethics statement

This study was carried out in accordance with the recommendations of Waseda University Academic Research Ethical Review Committee with written informed consent from all subjects. All subjects gave written informed consent in accordance with the Declaration of Helsinki. The protocol was approved by the Waseda University Academic Research Ethical Review Committee.

## Author contributions

IK designed the work and acquired, analyzed, and interpreted data for the work. IK drafted the work. HK substantially contributes to the design of the work and interpret data for the work. HK revised the draft critically for important intellectual content. IK and HK approves of the version to be published and agrees to be accountable for all aspects of the work in ensuring that questions related to the accuracy or integrity of any part of the work are appropriately investigated and resolved.

### Conflict of interest statement

The authors declare that the research was conducted in the absence of any commercial or financial relationships that could be construed as a potential conflict of interest.

## References

[B1] Allen M. Smallwood J. Christensen J. Gramm D. Rasmussen B. Jensen C. G.. (2013). The balanced mind: the variability of task-unrelated thoughts predicts error monitoring. Front. Hum. Neurosci. 7:743. 10.3389/fnhum.2013.00743 24223545 PMC3819597

[B2] Baer R. A. Smith G. T. Hopkins J. Krietemeyer J. Toney L. (2006). Using self-report assessment methods to explore facets of mindfulness. Assessment 13, 27–45. 10.1177/1073191105283504 16443717

[B3] Berkovich-Ohana A. Glicksohn J. Goldstein A. (2012). Mindfulness-induced changes in gamma band activity-implications for the default mode network, self-reference and attention. Clin. Neurophysiol. 123, 700–710. 10.1016/j.clinph.2011.07.048 21940201

[B4] Bixler R. D'Mello S. (2016). Automatic gaze-based user-independent detection of mind wandering during computerized reading. User Model User Adap. Inter. 26, 33–68. 10.1007/s11257-015-9167-1

[B5] Blanchard N. Bixler R. Joyce T. D'Mello S. (2014). Automated physiological-based detection of mind wandering during learning, in Intelligent Tutoring Systems. ITS 2014. Lecture Notes in Computer Science, eds Trausan-Matu S. Boyer K. E. Crosby M. Panourgia K. (Cham: Springer International Publishing), 55–60.

[B6] Braboszcz C. Delorme A. (2011). Lost in thoughts: neural markers of low alertness during mind wandering. Neuroimage 54, 3040–3047. 10.1016/j.neuroimage.2010.10.008 20946963

[B7] Burg J. M. Michalak J. (2010). The healthy quality of mindful breathing: associations with rumination and depression. Cogn. Ther. Res. 35, 179–185. 10.1007/s10608-010-9343-x

[B8] Christoff K. Gordon A. M. Smallwood J. Smith R. Schooler J. W. (2009). Experience sampling during fMRI reveals default network and executive system contributions to mind wandering. Proc. Natl. Acad. Sci. U.S.A. 106, 8719–8724. 10.1073/pnas.0900234106 19433790 PMC2689035

[B9] Damoiseaux J. S. Beckmann C. F. Arigita E. J. S. Barkhof F. Scheltens P. Stam C. J.. (2008). Reduced resting-state brain activity in the “default network” in normal aging. Cereb. Cortex 18, 1856–1864. 10.1093/cercor/bhm207 18063564

[B10] Drummond S. P. A. Walker M. Almklov E. Campos M. Anderson D. E. Straus L. D. (2013). Neural correlates of working memory performance in primary insomnia. Sleep 36, 1307–1316. 10.5665/sleep.2952 23997363 PMC3738039

[B11] Farley J. Risko E. F. Kingstone A. (2013). Everyday attention and lecture retention: the effects of time, fidgeting, and mind wandering. Front. Psychol. 4:619. 10.3389/fpsyg.2013.00619 24065933 PMC3776418

[B12] Forster S. Nunez Elizalde A. O. Castle E. Bishop S. J. (2015). Unraveling the anxious mind: anxiety, worry, and frontal engagement in sustained attention versus off-task processing. Cereb. Cortex 25, 609–618. 10.1093/cercor/bht248 24062316 PMC4318530

[B13] Hastie T. Tibshirani R. Friedman J. (2009). The Elements of Statistical Learning. 2nd Edn. Berlin: Springer.

[B14] Hoexter M. Q. Miguel E. C. Diniz J. B. Shavitt R. G. Busatto G. F. Sato J. R. (2013). Predicting obsessive-compulsive disorder severity combining neuroimaging and machine learning methods. J. Affect. Disord. 150, 1213–1216. 10.1016/j.jad.2013.05.041 23769292

[B15] Ito M. (2000). Mechanisms of motor learning in the cerebellum. Brain Res. 886, 237–245. 10.1016/S0006-8993(00)03142-5 11119699

[B16] Jensen C. G. Niclasen J. Vangkilde S. A. Petersen A. Hasselbalch S. G. (2016). General inattentiveness is a long-term reliable trait independently predictive of psychological health: danish validation studies of the mindful attention awareness scale. Psychol. Assess. 28, e70–e87. 10.1037/pas000019 26751089

[B17] Kabat-Zinn J. (2015). Mindfulness. Mindfulness 6, 1481–1483. 10.1007/s12671-015-0456-x

[B18] Killingsworth M. A. Gilbert D. T. (2010). A wandering mind is an unhappy mind. Science 330, 932–932. 10.1126/science.1192439 21071660

[B19] Kubicki S. Herrmann W. M. Fichte K. Freund G. (1979). Reflections on the topics: EEG frequency bands and regulation of vigilance. Pharmakopsychiatr. Neuropsychopharmakol. 12, 237–245. 10.1055/s-0028-1094615 223177

[B20] Kucyi A. Esterman M. Riley C. S. Valera E. M. (2016). Spontaneous default network activity reflects behavioral variability independent of mind-wandering. Proc. Natl. Acad. Sci. U.S.A. 113, 13899–13904. 10.1073/pnas.1611743113 27856733 PMC5137714

[B21] Maillet D. Rajah M. N. (2016). Assessing the neural correlates of task-unrelated thoughts during episodic encoding and their association with subsequent memory in young and older adults. J. Cogn. Neurosci. 28, 826–841. 10.1162/jocn_a_00935 26845110

[B22] Maillet D. Schacter D. L. (2016). From mind wandering to involuntary retrieval: age-related differences in spontaneous cognitive processes. Neuropsychologia 80, 142–156. 10.1016/j.neuropsychologia.2015.11.017 26617263 PMC4698179

[B23] Marchetti I. Van de Putte E. Koster E. H. W. (2014). Self-generated thoughts and depression: from daydreaming to depressive symptoms. Front. Hum. Neurosci. 8:131. 10.3389/fnhum.2014.00131 24672458 PMC3957030

[B24] Mason M. F. Norton M. I. Van Horn J. D. Wegner D. M. Grafton S. T. Macrae C. N. (2007). Wandering minds: the default network and stimulus-independent thought. Science 315, 393–395. 10.1126/science.1131295 17234951 PMC1821121

[B25] Menon V. (2011). Large-scale brain networks and psychopathology: a unifying triple network model. Trends Cogn. Sci. 15, 483–506. 10.1016/j.tics.2011.08.003 21908230

[B26] Mittner M. Boekel W. Tucker A. M. Turner B. M. Heathcote A. Forstmann B. U. (2014). When the brain takes a break: a model-based analysis of mind wandering. J. Neurosci. 34, 16286–16295. 10.1523/JNEUROSCI.2062-14.2014 25471568 PMC4252543

[B27] Mizuhara H. Yamaguchi Y. (2007). Human cortical circuits for central executive function emerge by theta phase synchronization. Neuroimage 36, 232–244. 10.1016/j.neuroimage.2007.02.026 17433880

[B28] Mrazek M. D. Franklin M. S. Phillips D. T. Baird B. Schooler J. W. (2013). Mindfulness training improves working memory capacity and gre performance while reducing mind wandering. Psychol. Sci. 24, 776–781. 10.1177/0956797612459659 23538911

[B29] Mwangi B. Tian T. S. Soares J. C. (2014). A review of feature reduction techniques in neuroimaging. Neuroinformatics 12, 229–244. 10.1007/s12021-013-9204-3 24013948 PMC4040248

[B30] Nolen-Hoeksema S. Wisco B. E. Lyubomirsky S. (2008). Rethinking rumination. Perspect. Psychol. Sci. 3, 400–424. 10.1111/j.1745-6924.2008.00088.x 26158958

[B31] Radloff L. S. (1977). The CES-D scale: a self-report depression scale for research in the general population. Appl. Psychol. Meas. 1, 385–401. 10.1177/014662167700100306

[B32] Raichle M. E. MacLeod A. M. Snyder A. Z. Powers W. J. Gusnard D. A. Shulman G. L. (2001). A default mode of brain function. Proc. Natl. Acad. Sci. U.S.A. 98, 676–682. 10.1073/pnas.98.2.676 11209064 PMC14647

[B33] Robertson I. H. Manly T. Andrade J. Baddeley B. T. Yiend J. (1997). “Oops!”: performance correlates of everyday attentional failures in traumatic brain injured and normal subjects. Neuropsychologia 35, 747–758. 10.1016/S0028-3932(97)00015-8 9204482

[B34] Sauseng P. Klimesch W. Schabus M. Doppelmayr M. (2005). Fronto-parietal EEG coherence in theta and upper alpha reflect central executive functions of working memory. Int. J. Psychophysiol. 57, 97–103. 10.1016/j.ijpsycho.2005.03.018 15967528

[B35] Schad D. J. Nuthmann A. Engbert R. (2012). Your mind wanders weakly, your mind wanders deeply: objective measures reveal mindless reading at different levels. Cognition 125, 179–194. 10.1016/j.cognition.2012.07.004 22857818

[B36] Scheeringa R. Bastiaansen M. C. M. Petersson K. M. Oostenveld R. Norris D. G. Hagoort P. (2008). Frontal theta EEG activity correlates negatively with the default mode network in resting state. Int. J. Psychophysiol. 67, 242–251. 10.1016/j.ijpsycho.2007.05.017 17707538

[B37] Schooler J. W. Smallwood J. Christoff K. Handy T. C. Reichle E. D. Sayette M. A. (2011). Meta-awareness, perceptual decoupling and the wandering mind. Trends Cogn. Sci. 15, 319–326. 10.1016/j.tics.2011.05.006 21684189

[B38] Schubotz R. I. von Cramon D. Y. (2003). Functional–anatomical concepts of human premotor cortex: evidence from fMRI and PET studies. Neuroimage 20(Suppl. 1), S120–S131. 10.1016/j.neuroimage.2003.09.014 14597305

[B39] Seeley W. W. Menon V. Schatzberg A. F. Keller J. Glover G. H. Kenna H.. (2007). Dissociable intrinsic connectivity networks for salience processing and executive control. J. Neurosci. 27, 2349–2356. 10.1523/JNEUROSCI.5587-06.2007 17329432 PMC2680293

[B40] Segal Z. V. Williams J. M. G. Teasdale J. D. (2002). Mindfulness-Based Cognitive Therapy for Depression: A New Approach To Preventing Relapse. New York, NY: Gilford Press.

[B41] Shin D.-J. Lee T. Y. Jung W. H. Kim S. N. Jang J. H. Kwon J. S. (2015). Away from home: the brain of the wandering mind as a model for schizophrenia. Schizophr. Res. 165, 83–89. 10.1016/j.schres.2015.03.021 25864955

[B42] Smallwood J. McSpadden M. Schooler J. W. (2007a). The lights are on but no one's home: meta-awareness and the decoupling of attention when the mind wanders. Psychon. Bull. Rev. 14, 527–553. 10.3758/BF03194102 17874601

[B43] Smallwood J. McSpadden M. Schooler J. W. (2008). When attention matters: the curious incident of the wandering mind. Mem. Cognit. 36, 1144–1150. 10.3758/MC.36.6.1144 18927032

[B44] Smallwood J. O'Connor R. C. Sudbery M. V. Obonsawin M. (2007b). Mind-wandering and dysphoria. Cogn. Emot. 21, 816–842. 10.1080/02699930600911531

[B45] Smallwood J. Schooler J. W. (2006). The restless mind. Psychol. Bull. 132, 946–958. 10.1037/0033-2909.132.6.946 17073528

[B46] Smallwood J. Schooler J. W. (2015). The science of mind wandering: empirically navigating the stream of consciousness. Annu. Rev. Psychol. 66, 487–518. 10.1146/annurev-psych-010814-015331 25293689

[B47] Stawarczyk D. Majerus S. Maquet P. D'Argembeau A. (2011). Neural correlates of ongoing conscious experience: both task-unrelatedness and stimulus-independence are related to default network activity. PLoS ONE 6:e16997. 10.1371/journal.pone.0016997 21347270 PMC3038939

[B48] Zavagnin M. Borella E. De Beni R. (2014). When the mind wanders: age-related differences between young and older adults. Acta Psychol. 145, 54–64. 10.1016/j.actpsy.2013.10.016 24291121

[B49] Zich C. Schweinitz C. Debener S. Kranczioch C. (2015). Multimodal evaluation of motor imagery training supported by mobile EEG at home: A case report, in Systems, Man, and Cybernetics (SMC), 2015 IEEE International Conference on. (Hong Kong), 3181–3186.

